# Repeated quantitative measurements of *De Novo* synthesis of albumin and fibrinogen

**DOI:** 10.1371/journal.pone.0174611

**Published:** 2017-03-28

**Authors:** Gabriel Dumitrescu, Andras Komaromi, Olav Rooyackers, Maria Klaude, Christina Hebert, Jan Wernerman, Åke Norberg

**Affiliations:** 1 Department of Anesthesia and Intensive Care Medicine at Karolinska University Hospital, Stockholm, Sweden; 2 Department of Clinical Science, Intervention and Technology CLINTEC, Karolinska Institutet, Stockholm, Sweden; Kermanshah University of Medical Sciences, ISLAMIC REPUBLIC OF IRAN

## Abstract

The possibility of using two different isotopomers, for the incorporation of isotopically labeled amino acids, was explored to enable longitudinal studies of de novo synthesis of two export liver proteins, albumin and fibrinogen. The agreement of the synthesis rates between the two different labels was evaluated along with the reproducibility of repeated experiments using different time intervals. Healthy volunteers were studied in a standardized fed state. Protocol A (n = 10) involved two measurements 48 hours apart. Protocol B (n = 6) involved three measurements at baseline and five hours and then seven days after the initial measurement. De novo synthesis of albumin and fibrinogen by the incorporation of D_5_-phenylalanine or D_8_-phenylalanine were measured using the flooding dose technique. Albumin and fibrinogen were isolated from plasma using standard techniques. Fractional and absolute synthesis rates were calculated. Repeated measurements employing the two isotoptomers showed good agreement for albumin fractional synthesis rate after 48 hours (p = 0.92) and after 7 days (p = 0.99), with a coefficient of variation of 5.9% when using the same isotopic label. For fibrinogen, the coefficient of variation for the fractional synthesis rate employing the same isotopic label was 16.6%. Repeated measurements after 48 hours and seven days showed less agreement although there was no statistical difference (P = 0.32 and P = 0.30 respectively). Repeated measurement after five hours showed a statistical significant difference for the fractional synthesis rate of fibrinogen (p = 0.008) but not for albumin (p = 0.12). Repeated measurements of albumin de novo synthesis more than 48 hours apart show acceptable agreement using either one or two different isotopic labels. For fibrinogen the larger intra-individual scatter necessitates larger study groups to detect changes in longitudinal studies. Repeated measurements within 48 hours need to be validated further.

## Introduction

The changes in plasma concentrations of several liver export proteins in relation to surgical procedures, inflammation and infectious states are well known. However, the mechanisms behind these changes are less well understood. Altered synthesis rates or degradation rates are obvious possibilities as well as changes in plasma volume and a redistribution of the proteins into different compartments.

Studies of albumin demonstrate an almost 50% reduction in plasma concentration within a few hours after the start of a major surgical procedure, attributable to a number of interventions and physiological perturbations [1, 2]. Still, the albumin synthesis rate is unaltered on postoperative day two when compared to the preoperative rate, despite the dramatic decrease in plasma concentration [3]. A similar low plasma concentration associated with acute inflammation/infection is accompanied by an elevated albumin synthesis rate [1], and a similarly high synthesis rate is also seen in critically ill patients in the intensive care unit (ICU) [4–6]. For another liver export protein, fibrinogen, a higher than normal plasma concentration is seen in chronic inflammation, which is associated with an elevated synthesis rate [7]. In liver failure, a normal synthesis rate is reported though there is a lower than normal plasma fibrinogen concentration [8]. Following surgical liver resections, a decrease in plasma fibrinogen concentration is seen, followed by a rapid recovery to a normal level [9]. However, in that situation, data on fibrinogen synthesis rate is not available.

To be able to further study these processes perioperatively and during critical illness, access to techniques that enable not only determination of plasma concentrations, but also de novo synthesis rates are necessary. In particular, longitudinal studies involving the same subjects and repeated measurements are attractive. However, repetitive sampling with short time intervals may be problematic using techniques involving the incorporation of isotopic-labeled amino acids into proteins. This problem was demonstrated for both albumin and for mixed proteins in the liver [10]. A second measurement within a short period may be compensated for by increasing the dose of the isotopically labeled substrate, but the altered baseline induced by the first measurement will always make the second measurement more problematic [11]. Therefore, the introduction of a second isotopic label may be an attractive solution, as the isotopic baseline of the second isotope will not be changed by the first measurement.

In order to explore the possibility of repeated measurements of de novo synthesis of two liver export proteins, albumin and fibrinogen, by incorporation of two different isotopic labels of phenylalanine, two different study protocols were performed. The goal was to demonstrate equality (non-inferiority) between the two isotopic labels in two measurements 48 hours apart with administration of the two tracers in random order. In addition, a five hour interval between measurements employing the two tracers was studied, in combination with a repeated measurement of the initial tracer seven days later to establish the variability of repeated measurements. All studies were performed in a standardized fed state.

## Materials and methods

### Protocol

Healthy volunteers were recruited from a register of interested individuals kept by the departmental research centre. They were screened during May-October 2014, and included into the study during the same time period. The subjects were investigated using two different protocols. The protocols were approved by the Regional Ethics Committee in Stockholm, Sweden, and were in accordance with the Helsinki Declaration of 1975. The subjects gave written informed consent both orally and in writing after being informed about the study protocol. The study was prospectively registered at ANZCTR, ID ACTRN12614000541606.

Group A was studied using two time points 48 hours apart, and group B was using three time points, at basal, five hours, and seven days afterwards. On each occasion the subjects were without caloric intake for more than 4 hours, and then a standardized snack of a cheese sandwich and a mug of yoghurt were given 30 minutes before start of the study protocol.

The protocol used on each study occasion was identical, except for the isotoptomers given. After insertion of two venous accesses in antecubital veins, D_5_-phenylalanine or D_8_-phenylalanine (45mg/kg, 10 MPE [molar percent excess], > 98% purity) of a 2% (2g/100mL saline) phenylalanine solution was infused intravenously for 10 minutes. For the infusion after seven days a 20 MPE solution was used. Thereafter venous blood samples were obtained just before and at 5, 10, 15, 30, 40, 50, 60, 70, and 90 minutes after the start of the tracer phenylalanine infusion. The samples, collected into EDTA vials and kept on ice, were centrifuged at 2,500 xG at 4^°^C and the plasma fraction was then stored at -80^°^C pending analysis.

In protocol A (n = 10), the goal was to compare the synthesis rates of albumin and fibrinogen as measured by the incorporation of the two different isotopic tracers of phenylalanine, D_5_-phenylalanine and D_8_-phenylalanine, administered blindly in random order, 48 hours apart. In protocol B (n = 6) the goal was to compare the synthesis rates of albumin and fibrinogen seven days apart as measured by the incorporation of D_5_-phenylalanine, when the influence of the first experiment on the second experiment was considered minimal. Additionally the synthesis rates of albumin and fibrinogen as measured by the incorporation of D_8_-phenylalanine were compared five hours after the initial measurement by the incorporation of D_5_-phenylalanine.

### Analyses

For the calculation of the fractional synthesis rate (FSR), the enrichment of D_5-_ and D_8-_ phenylalanine in the plasma free phenylalanine pool and in circulating albumin and fibrinogen were measured.

For the free plasma enrichment, proteins were precipitated with sulfosalicylic acid and the supernatant was purified using ion exchange resin (AG-50W-X8, Bio-Rad, Solna Sweden). D_5_-, D_8_- and unlabeled phenylalanine were measured as their *tert-*butyldimethylsilyl derivative using gas chromatography- mass spectrometry (GC-MS; Agilent 5975C, Agilent, Kista, Sweden). For derivatization, the dried sample was incubated with acetate/ *N*-Metyl-N-(tert-butyldimetylsilyl) trifluoroacetamide (50/50) for 60 min at 60^°^C. Because about 50% of D_8_-phenylalanine in plasma occurs as D_7_-phenylalanine due to in vivo conversion the sum of D_7-_ and D_8_-phenylalanine was used for further calculations as suggested [12, 13].

For the enrichment measurements of albumin and fibrinogen, these two proteins were isolated from plasma samples as previously described [8, 14]. In short, albumin was isolated by ethanol extraction from trichloroacetic acid-precipitated proteins. After several wash steps, albumin was hydrolyzed using 6 M hydrochloric acid (HCL) (110^°^C for 24 hours). Fibrinogen was isolated by dissolving several times in sodium citrate, followed by precipitation with L-ammonium sulfate. Finally fibrinogen was precipitated using perchloric acid and hydrolyzed using 6 M HCL (110^°^C for 24 hours). Following hydrolyzation, the enrichment of phenylalanine from both proteins was analyzed after conversion of phenylalanine to phenylethylamine using tyrosine decarboxylase and extraction with diethyl ether [15]. The conversion of phenylalanine to phenylethylamine makes it possible to extract it from other amino acids and to eliminate the background noise almost to zero and thereby obtaining an optimal signal to noise ratio. To enable measurements of low enrichments on a GC-MS (0–0.26MPE), enrichments of D_5-_, D_7-_ and D_8-_phenylalanine in the proteins were estimated from the measurements of mass ratios of m+5/m+2, m+7/m+2 and m+8/m+2 of phenylethylamine and compared with standard curves of known phenylalanine enrichments, which are treated and measured similarly as the protein samples [7, 15]. The contribution of D5-phenylalanine to the D7-phenylalanine was very low and since the two tracers were not given simultaneously this contribution was ignored. FSRs were calculated by dividing the increment of the enrichment in the protein by the area under the curve for the precursor enrichment in plasma. For D_8_-phenylalanine the sum of D_7-_ and D_8-_phenylalanine was used.

FSR: [P_t2_-P_t1_]/AUC ∙ 1440∙ 100.

In which [P_t2_-P_t1_] is the delta enrichment in the protein; AUC is the area under the curve enrichment of the precursor (adjusted for the secretion time of the protein); the factors 1440 and 100 are for calculating FSR as %/24 hours.

Concentrations of albumin and fibrinogen were analyzed in the hospitals routine laboratory using standard photospectrometric analyzes with an albumin bromocresol purple (BCP) assay on a Cobas c701 chemistry analyzer(Roche Diagnostics, Stockholm, Sweden) and a Clauss fibrinogen assay on a CS-2100i blood coagulation analyzer (Sysmex, Kungsbacka, Sweden). Absolute synthesis rates (ASR) of albumin and fibrinogen were calculated by multiplying the FSR with the total content of albumin/fibrinogen in the plasma. The plasma volume was calculated using standard anthropometric estimates [16].

### Statistics

Baseline characteristics are reported as means ± standard deviation or as medians (range) as appropriate. In protocol A Wilcoxon’s matched pairs test was used. The coefficients of variation between two measurements were determined using Dahlberg analyses [17]. In study B (three time points) Friedman’s repeated measures analysis of variance (ANOVA) was used with Dunn’s post-hoc testing of differences. Prism 6 (GraphPad Software Inc, La Jolla, CA) was used for the statistical analysis.

The number of subjects included in study A made it possible to detect or exclude a difference of > 1 standard deviation with 80% statistical power. The number of subjects in study B was chosen for calculation of the coefficient of variation (CV) of repeated measurements. The 5h measurements were added to explore the possible difference of a short term repeat as hypothesis generating pilot for a larger study, which was abolished.

## Results

The characteristics of the healthy volunteers are given in Table 1. In addition characterization and some results related to gender are given in the electronic supplement in S1 Table.

**Table 1:** Anthropometric and laboratory data.

	Study A, n = 10	Study B, n = 6
**Gender (Female:Male)**	4: 6	3:3
**Age (years)**	40.6 ± 17.8 ^a^	36.8 ± 14.9 ^b^
**Weight (kg)**	82.6 ± 12.5	78.5 ± 11.5
**Heigth (cm)**	177 ± 10	175 ± 8
**Plasma volume (L)**	2.97 ± 0.41	2.86 ± 0.34
**P-Albumin (g/L)**	35.6 ± 2.2	37.0 ± 1.7
**P-Fibrinogen (g/L)**	2.54 ± 0.64	2.41 ± 0.24

Data are given as means ± standard deviation

^a^ 43.5 (20–67), median (range)

^b^ 35 (20–61) median (range)

**Fig 1:**
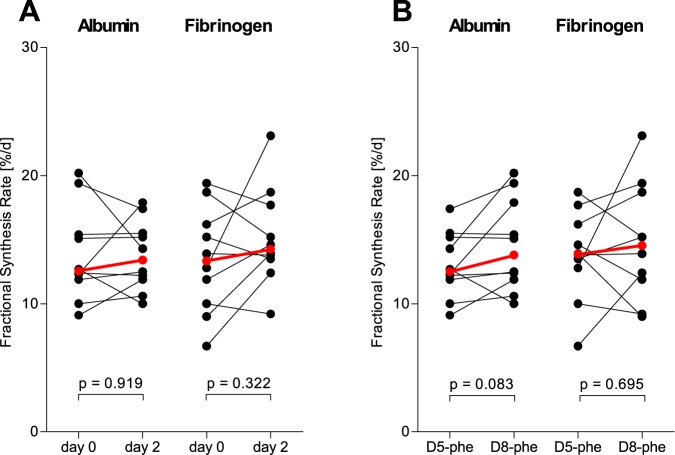
Fractional synthesis rates for albumin and fibrinogen in healthy volunteers (n = 10), measured by the incorporation of D5-phenylalanine or D8-phenylalanine administered 48 hours apart in random order in a standardized fed state. (A) The time points and (B) the pattern of the two isotopic tracers used to determine synthesis rates. P-values correspond to comparison of the two measurements by Wilcoxon’s t-test for paired observations. The red and bolded symbols represent the medians.

**Fig 2:**
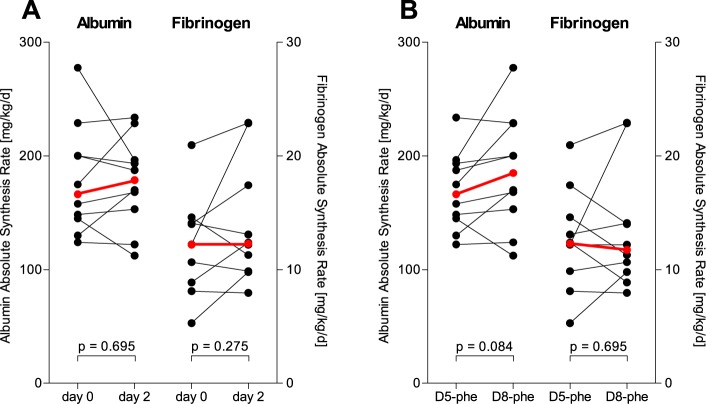
Absolute synthesis rates for albumin and fibrinogen in healthy volunteers (n = 10), measured by the incorporation of D5-phenylalanine or D8-phenylalanine administered in random order 48 hours apart in a standardized fed state. (A) The time points and (B) the pattern of the two isotopic tracers used to determine synthesis rates. P-values correspond to comparison of the two measurements by Wilcoxon’s t-test for paired observations. The red and bolded symbols represent the medians.

**Fig 3:**
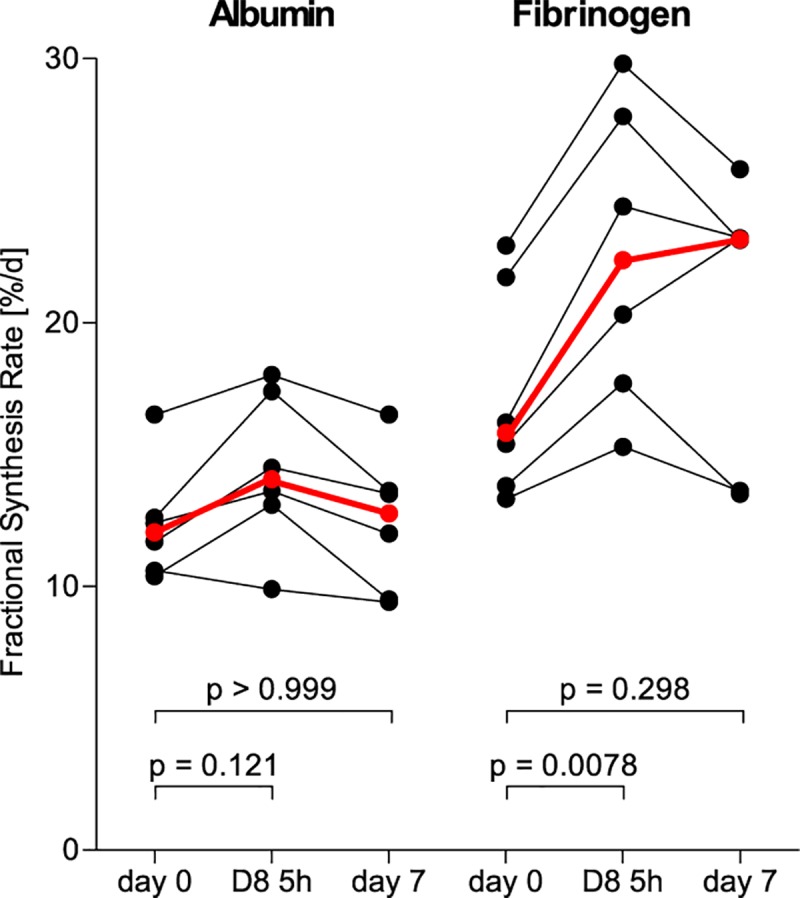
Fractional synthesis rates for albumin and fibrinogen in healthy volunteers (n = 6), measured by the incorporation of D5-phenylalanine at baseline and at day 7, or by D8-phenylalanine at 5 hours after baseline. The red and bolded symbols represent the medians. Friedman’s repeated measures ANOVA revealed significant differences over time for both albumin (p = 0.0275) and fibrinogen (p = 0.0120). P-values in the figure correspond to Dunn’s post-hoc testing.

**Fig 4:**
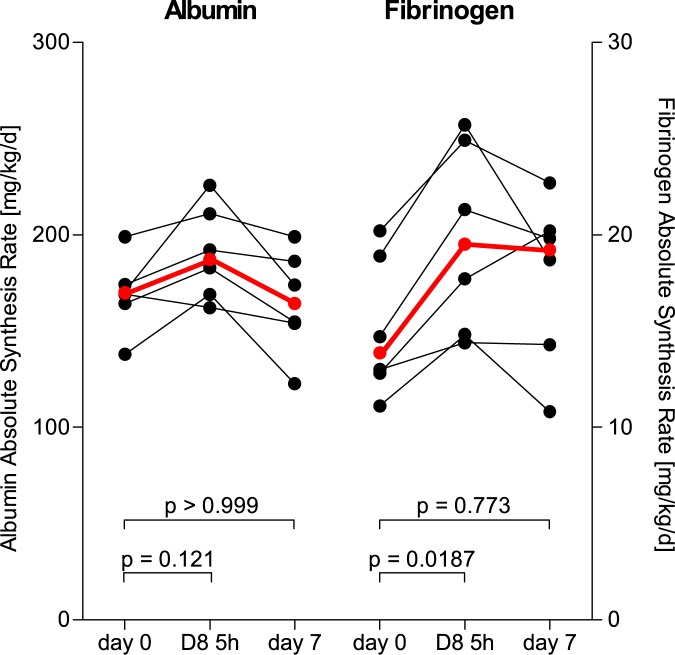
Absolute synthesis rates for albumin and fibrinogen in healthy volunteers (n = 6), measured by the incorporation of D5-phenylalanine at baseline and at day 7, or by D8-phenylalanine at 5 hours after baseline. The red and bolded symbols represent the medians. Friedman’s repeated measures ANOVA revealed significant differences over time for both albumin (p = 0.0275), and fibrinogen (p = 0.0289). P-values in the figure correspond to Dunn’s post-hoc testing.

The results of the albumin and fibrinogen FSRs for measurements taken 48 hours apart are illustrated in Fig 1. In Fig 2, the albumin and fibrinogen ASRs are illustrated, where the plasma volumes for the subjects were calculated using anthropometric data. No group differences between days or tracers reached statistical significance. When investigated seven days apart by the same isotopic phenylalanine tracer (D_5_-phenylalanine), both FSRs and ASRs of albumin and fibrinogen were similar as shown in Figs 3 and 4, respectively. The coefficients of variation as calculated from these two measurements were 5.9% for albumin and 16.6% for fibrinogen. When measured five hours apart by the incorporation of two different isotopic labels of phenylalanine, there were increases in both synthesis rates for both albumin and fibrinogen as shown in Figs 3 and 4. Underlying data for the calculations of the fractional synthesis rates of albumin and fibrinogen are given in the electronic supplement in S2 Table.

## Discussion

The goal of the present study was to use two different isotopic labels of phenylalanine to determine the synthesis rates of the liver export proteins albumin and fibrinogen. The results show that measurements 48 hours or more apart give comparable results on a group level, while measurements five hours apart show interference that make this shorter period unsuitable for repeated measurements. The similar patterns of results for the two different plasma proteins suggests there is a sufficient basis for this conclusion. Whether a time period somewhere in between 5 and 48 hours would be sufficient to allow for reliable repeated measurements is outside the scope of this study. In longitudinal studies with repeated measurements, an interval of more than 48 hours is recommended, in particular when more than two measurements are planned. For future studies, there may be a choice between less frequent sampling, or to investigate parallel groups at different times. The latter alternative will of course necessitate a much larger number of involved subjects. The coefficient of variation for repeated measurements one week apart is most likely attributable to a combination of physiologic variability together with analytical and protocol related factors, such as sample preparation, response to feeding etc. This underlines the need to standardize these factors as much as possible when repeated measurements for paired comparisons are used. The inter-individual variability has been highlighted by several investigators related to feeding status, body composition, age, gender, etc [18–20]. The influence of gender in a post hoc analysis of data in the present study, presented in S1 Table, showed a higher value of P-fibrinogen in females, accompanied by a higher absolute synthesis rate. Among possible confounders, the females were older [21–23] and had a lower hematocrit, while body mass index and plasma volume per kg body weight were similar in both gender groups.

This study also has provided additional important information on the two plasma proteins tested. Our working group has published repeatedly on the albumin synthesis rates in healthy individuals as well as in different patient cohorts [1, 3, 10, 14, 24–26]. The FSRs have been reported as being between 6 and 10% per 24 hours. This has been in the post-absorptive state, except for ICU patients who were continuously fed [4, 5]. Here, the FSRs for albumin in healthy subjects were around 15% per 24 hours. The obvious protocol difference is the standardized feeding used in the present protocol. As demonstrated by Caso et al, feeding is associated with a 50% increase in albumin synthesis rate, as result of a protein meal and/or a combined meal [27]. From these results, it is obvious that the state of feeding is an important consideration when the synthesis rate of albumin is measured. The slight difference in synthesis rates between the present study and Caso et al, may be attributable to differences in the amount, content, and timing of feeding. The same argument may be applicable to the synthesis rate of fibrinogen, which Caso et al reported as showing a similar effect in another publication [22]. In general the amino acid availability is shown to be a major determinant in the regulation of protein metabolism [28].

The flooding dose technique was used to guarantee that the use of plasma isotope enrichment as a substitute for the intracellular amino acid precursor pool in the hepatocytes is a fair assumption. A constant infusion approach would be considerably more problematic, as demonstrated by Caso et al for muscle tissue [29], where access to tissue samples is a necessary condition to be able to assess the effects of feeding by the constant infusion approach. As the effect of feeding was larger rather than smaller than anticipated, our choice to use the flooding technique was reassuring. The alternative would be to establish a nutritional steady state by a constant intravenous infusion, during such condition plasma enrichment is a reliable reflection of the intracellular precursor [30, 31].

The assessment of the fibrinogen synthesis rate in the present study is a needed addition to the small number of publications detailing the de novo synthesis of fibrinogen in humans [7, 8, 19, 22, 23, 32–34]. The quantitative values of our results are in agreement with these prior publications. The same issue for albumin also applies for fibrinogen, wherein the effect of feeding will be an important consideration in future study protocols.

The strengths of the present study are the study protocols with a standardized relation to food intake and the well-separated intervals between study time points. The limitations are the relatively small number of subjects included and the introduction of the fed state as a standardized reference without a more thorough characterization. In regards to the small number of subjects, however, it can be argued that it is an ethical issuse to use more subjects than necessary to answer the question put forward. Another limitation is of course the span of time between the 5 and 48 hour study time points, which is not covered in the present protocols. For our purposes, however, in the planned clinical studies a shorter interval will not be used.

In conclusion the use of different isotopic tracers of phenylalanine to measure the synthesis rate of albumin and fibrinogen within a time interval of five hours was not advisable, while an interval of 48 hours between measurements gave reliable results on a group level. In addition, the study confirmed earlier reports of the importance to properly standardize the state of feeding when de novo synthesis of liver export proteins like albumin and fibrinogen are studied.

## Supporting information

S1 Table Anthropometry and plasma protein parameters according to gender. For parameters that were repeatedly measured (n = 2–6), only the mean value for each individual subject was used in the calculations.
(PDF)

S2 Table Enrichment data used for the calculation of FSR of albumin and fibrinogen. (XLSX)
