# Supplementary material for: Repeated quantitative measurements of De Novo synthesis of albumin and fibrinogen
Source: PLoS One. 2017 Mar 28;12(3):e0174611. doi: 10.1371/journal.pone.0174611 (PMC5370154; doi:10.1371/journal.pone.0174611)
Supplement: S1 Table — For parameters that were repeatedly measured (n = 2–6), only the mean value for each individual subject was used in the calculations. (PDF) [file pone.0174611.s001.pdf]

Table. Anthropometry and plasma protein parameters according to gender.
For parameters that were repeatedly measured (n= 2-6), only the mean value for each individual subject was used in the calculations.

	Males (n=9)	Females (n=7)	p
**BMI (kg*m-2)**	26.4 ± 4.0	25.9 ± 3.8	0.805
**Hct (fraction)**	0.44 ± 0.02	0.39 ± 0.02	0.0004
**Plasma volume (L)**	3.15 ± 0.33	2.65 ± 0.20	0.0032
**Plasma volume (L/kg)**	36.6 ± 3.3	36.2 ± 3.4	0.821
**P-Albumin (g/L)**	37.0 ± 1.2	35.0 ± 2.5	0.053
**P-Fibrinogen (g/L)** [1]	2.2 (1.9 - 2.6)	2.7 (2.2 - 3.7)	0.020
**Albumin ASR (mg/kg/d)**	174 ± 30	180 ± 40	0.712
**Fibrinogen ASR (mg/kg/d)**	12.6 ± 4.7	17.5 ± 3.3	0.034
**Albumin FSR (%/d)**	13.0 ± 3.1	14.1 ± 2.0	0.437
**Fibrinogen FSR (%/d)**	15.4 ± 4.9	17.8 ± 4.6	0.345

Data are presented as mean ± SD and the p-value represents t-test for independent groups (gender), with an exception for P-fibrinogen where normality of data was rejected according to Shapiro-Wilk's W-test of normality. Therefore Mann-Whitney U-test was used for P-fibrinogen (denoted [1]), and values presented as median (range).
BMI body mass index, Hct hematocrit, ASR absolute synthesis rate, FSR fractional synthesis rate.
